# The role of dysregulated ghrelin/LEAP-2 balance in eating disorder: a translational study in anorexia nervosa

**DOI:** 10.1192/j.eurpsy.2023.290

**Published:** 2023-07-19

**Authors:** C. Tezenas du Montcel, P. Duriez, J. Cao, N. Ramoz, O. Viltart, P. Gorwood, V. Tolle

**Affiliations:** 1 Université Paris Cité UMR-S 1266, Institut de Psychiatrie et Neurosciences de Paris, 75014 Paris; 2 Clinique des Maladies Mentales et de l’Encéphale, GHU Paris Psychiatrie et Neurosciences Hôpital Sainte Anne, F-75014 Paris; 3 Université Paris Cité UMR-S 1266 INSERM, Institut de Psychiatrie et Neurosciences de Paris - Inserm U1266, 75014 Paris; 4 Clinique des Maladies Mentales et de l’Encéphale, GHU Paris Psychiatrie et Neurosciences Hôpital Sainte-Anne, F-75014; 5 Université Paris Cité, UMR-S 1266 INSERM, Institut de Psychiatrie et Neuroscience de Paris, 75014; 6 Université Paris Cité, UMR-S 1266 INSERM, Institut de Psychiatrie et Neuroscience de Paris, 75014 Paris; 7 Faculté de Lille, Faculté des Sciences et Technologies, SN4, F-59650 Villeneuve d’Ascq; 8 1Université Paris Cité, UMR-S 1266 INSERM, Institut de Psychiatrie et Neuroscience de Paris, 75014 Paris; 9 Clinique des Maladies Mentales et de l’Encéphale, GHU Paris Psychiatrie et Neurosciences, Hôpital Sainte-Anne, F-75014 Paris, France

## Abstract

**Introduction:**

Anorexia nervosa (AN) is a complex psychiatric disorder characterized by a persistant decrease in food intake leading to dramatic weight loss and energy deficit. The ghrelin system is a key regulator of appetite and food intake across species. LEAP-2, a recently discovered ghrelin antagonist, appears to be up-regulated in obesity and opposes to the orexigenic drive of ghrelin. The evolution of LEAP-2 levels could be an interesting insight to reflect the regulation of appetite in eating disorders such as anorexia nervosa (AN).

**Objectives:**

We tested this hypothesis and here provide the first study exploring the ghrelin and LEAP-2 regulation in long-term food restriction followed by refeeding in both mice and patients suffering from AN.

**Methods:**

Using a translational strategy, we compared the regulation of ghrelin and LEAP-2 concentrations in blood during food restriction and after refeeding i/ in female mice exposed to a 14 days protocol combining quantitative food restriction and running wheel activity followed by 10 days of progressive refeeding; ii/ in an ongoing longitudinal study of patients with AN evaluated before and after refeeding (n=30) as well as 6 months after hospital discharge to evaluate if the weight gain was stable (n=7) or unstable (n=10). Plasma concentrations of ghrelin and LEAP-2 were measured with selective immunoassays.

**Results:**

Long-term food restriction in mice was associated with increased ghrelin (p<0.001) and decreased LEAP-2 concentrations (p=0.006) compared to *ad libitum* fed controls. Refeeding led to a decrease in ghrelin (p<0.01) and increase in LEAP-2 concentrations (p<0.01). Patients with AN displayed increased ghrelin levels (p<0.01) but also higher LEAP-2 concentrations on admission than after refeeding (p=0.04). LEAP-2 decreased with refeeding. On 17 patients re-evaluated 6 months after discharge, patients with unstable weight gain exhibited a greater decrease of LEAP-2 concentrations during refeeding compared to patient with stable weight gain (p=0.02). Decreasing LEAP-2 concentrations was able to predict a negative outcome (i.e. unstable weight gain) in 80% of the cases.

**Conclusions:**

We provide evidence that the ghrelin/LEAP-2 system is not regulated according to the nutritional status in AN as it is in the case of a physiological adaptation to food restriction. Results from an ongoing longitudinal study exploring remission in AN suggest that the evolution of LEAP-2 concentrations during refeeding is opposed to data from preclinical model and could give new insights on the outcome of weight gain in AN.

**Disclosure of Interest:**

None Declared

